# Instrumentation in Developing Chlorophyll Fluorescence Biosensing: A Review

**DOI:** 10.3390/s120911853

**Published:** 2012-08-29

**Authors:** Arturo A. Fernandez-Jaramillo, Carlos Duarte-Galvan, Luis M. Contreras-Medina, Irineo Torres-Pacheco, Rene de J. Romero-Troncoso, Ramon G. Guevara-Gonzalez, Jesus R. Millan-Almaraz

**Affiliations:** 1 Biosystems Engineering CA, Postgraduate Study Division, Engineering Faculty, Autonomous University of Queretaro, Cerro de las Campanas St., Querétaro, 76010, Qro., Mexico; E-Mails: aafernandez@hspdigital.org (A.A.F.-J.); cduarte20@alumnos.uaq.mx (C.D.-G.); mcontreras@hspdigital.org (L.M.C.-M.); irineo.torres@uaq.mx (I.T.-P.); ramon.guevara@uaq.mx (R.G.G.-G.); 2 HSPdigital-CA Mecatronics, Engineering Faculty, Autonomous University of Queretaro, Campus San Juan del Rio, 249 Rio Moctezuma St., San Juan del Rio, 76807, Qro., Mexico; E-Mail: troncoso@hspdigital.org; 3 Faculty of Physics and Mathematics, Autonomous University of Sinaloa, Universitarios Blvd., De las Americas Ave., Cd. Universitaria, Culiacan, 80000, Sinaloa, Mexico

**Keywords:** fluorescence, chlorophyll, photosynthesis, instrumentation, LED, laser, Kautsky

## Abstract

Chlorophyll fluorescence can be defined as the red and far-red light emitted by photosynthetic tissue when it is excited by a light source. This is an important phenomenon which permits investigators to obtain important information about the state of health of a photosynthetic sample. This article reviews the current state of the art knowledge regarding the design of new chlorophyll fluorescence sensing systems, providing appropriate information about processes, instrumentation and electronic devices. These types of systems and applications can be created to determine both comfort conditions and current problems within a given subject. The procedure to measure chlorophyll fluorescence is commonly split into two main parts; the first involves chlorophyll excitation, for which there are passive or active methods. The second part of the procedure is to closely measure the chlorophyll fluorescence response with specialized instrumentation systems. Such systems utilize several methods, each with different characteristics regarding to cost, resolution, ease of processing or portability. These methods for the most part include cameras, photodiodes and satellite images.

## 1. Introduction

Plants are among the most evolved beings on the planet; for example, unlike humans they are capable of producing their own nourishment through the process of photosynthesis. During this process sunlight energy is stored, and subsequently, in conjunction with water and carbon dioxide, forms carbohydrates and expels oxygen into the atmosphere [1]. However, plants cannot assimilate all the stored energy and approximately 5–10% of the total absorbed energy is accumulated as dry matter [2]. Consequently, plants are considered to be low-efficiency organisms. The excess energy is absorbed by the leaves must be dissipated through different paths involving thermal dissipative process, fluorescence emissions and photochemistry [3]. These losses can be utilized to research certain physiological behaviors in plants, such as photochemical and non-photochemical quenching.

Chlorophyll fluorescence is the red and far-red light emitted by photosynthetic tissue when it is excited by a light source [4]. Chlorophyll measurement is a non-invasive method for imaging photosynthetic fluxes because fluorescence depends directly on photosynthetic activity and it can be inferred through this [5–7].

This article reviews many applications in the measurement of fluorescence, most notably the detection of plant stress factors, with the objective of improving comfort conditions for the plant and providing higher production rates [8–11]. There are many factors that can produce plant diseases like stress caused by the environmental factors, among others [12]; however, these are not the only applications. Recently there has been climate impact research utilizing chlorophyll fluorescence analysis, along with satellite images [13] and manual measurement systems [14].

In order to develop instrumentation systems for measuring chlorophyll fluorescence, it is necessary to merge electrical engineering and plant biology. In this review, one vertex between the respective fields of electrical engineering and plant biosystems, is examined, especially the instrumentation when applied to the measurement of chlorophyll fluorescence. This phenomenon is common in some algae, bacteria and especially plants.

There are even some applications of chlorophyll fluorescence that focus on the quality, chemistry or physical characteristics of fruits; for instance, there are destructive methods which are commonly used in the process of detecting anthocyanin or flavonoids in specific fruits [1]. With chlorophyll fluorescence methods, this can be avoided. Regarding quality, there is research focused on plant nutrition characteristics to determine the types of elements that are beneficial for the plant and in what amounts [15,16]. Another important physical characteristic is the measurement of biomass, because it can be reflected directly in agricultural production [16], even in climate research through this characteristic [13].

None of the aforementioned techniques can be carried out without the appropriate instrumentation or equipment. The procedure proposed is made up of two main parts: the first being the chlorophyll excitation, for which there are active and passive methods. The active methods use lamps [17–19], LEDs [6,9,20–25], or lasers [16,26,27] to excite the chlorophyll before beginning the measurements. The passive methods takes advantage of solar radiation to achieve the same goal [28,29]. The second part of the procedure is the measurement of the chlorophyll fluorescence. This can include several methods, each with different characteristics such as cost, resolution, ease of processing or portability. These characteristics primarily take into account the cameras [19,30], photodiodes [6,21], optical fiber [31] and satellite images [13] used during the process.

This article is both an update and overarching review regarding the instrumentation and sensing methods for measuring chlorophyll fluorescence; the objective is to provide a panoramic view of this field of research to aid in future developments and research projects. The long term objective of this review is to provide useful information and key data: primarily, the wavelength, intensity, and operating conditions that a given source of active or passive chlorophyll excitation requires. Next, the proper handling of the sample for correct measurement is reviewed. After that, the connections, methods, and electronic devices to maximize fluorescence response acquisition are described. Finally, data processing of the fluorescence response which includes filters, algorithms and equations, is examined.

## 2. Evolving Knowledge of Chlorophyll Fluorescence

Hans W. Kautsky and his collaborator, A. Hirsh, discovered a time dependent fluorescence when they observed an increase in fluorescence intensity when dark-adapted photosynthetically active samples were illuminated [32]. This phenomenon is also known as fluorescence transient, OJIP curve, fluorescence induction, fluorescence decay or the Kautsky effect.

All plants, from higher specimens to unicellular green algae, possess a chloroplast with thylakoid membranes containing photosystems I (PSI) and II (PSII), which are connected to each other by the cytochrome b_6_f complex. PSI is capable of producing fluorescence, but in very small quantities which are negligible. PSII is the only viable contributor to variable fluorescence [20,33].

PSII consists of pigments and proteins in the thylakoid membrane of chloroplasts, which is the major target of many environmental stresses [34]. Based on this, chlorophyll fluorescence is widely used to determine the quality of growth conditions particularly in precision agriculture, where the parameters can be manipulated to improve the comfort conditions of plants.

Recent research suggests that the methods to dissipate excessive sunlight energy can be considered as a slowly reversible, protective mechanism, because inactive PSII complexes can act as protective shields [35]. Therefore, these mechanisms decrease the photochemical efficiency of PSII by interrupting the equilibrium between the radical pair and excitons in the light-harvesting antenna [36]. Consequently, photosynthetic energy harvesting is reduced and photon re-emitting by fluorescence is increased. Based in these facts, many techniques have been developed in the measurement of fluorescence for the improvement of comfort conditions in the plant and have increased production rates through the early detection of stress conditions [8–11].

**Figure 1.**
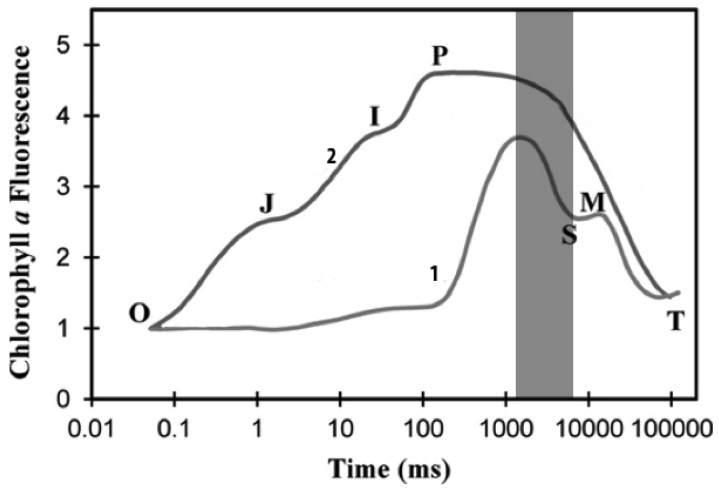
Chlorophyll *a* fluorescence induction transient, wavelength of excitation 650 nm and (1) 32 and (2) 3,200 μmol photons m^−2^ s^−1^ at the leaf surface respectively (modified from [33]).

In order to make accurate measurements of chlorophyll fluorescence, it is necessary to obtain the chlorophyll a fluorescence induction transient. This process is divided into two parts: the fast phase, which occurs within a second is labeled OJIP as shown in Figure 1, where O is the origin, P is the peak, and the two intermediate states are J-I [33]. This phase can be divided into two sub-phases; (1) the photochemical phase that consists of O-J, and is directly dependant on the intensity of the excitation light; and (2) the nonphotochemical phase that consists of the J-I-P transient and is dominated by mechanisms for the excitation-dependent thermal dissipation of energy from PSII [37,38].

In this part, the main characteristics of the OJIP curve are described. Due to the fact that F_O_ and F_M_ are the most common parameters, these can be translated to O and P, respectively. In normal non-stressed plant leaves, the ratio of F_M_/F_O_ is a constant high value [39]. There is another parameter called the variable fluorescence, F_V_, which is the difference between F_M_ and F_O_ as shown in Equation (1). The ratio shown in Equation (2) is related to the maximum quantum yields of primary PSII photochemistry [37,40,41]. The relative variable fluorescence, V(t) at time t is a parameter frequently used in graphical representations of fluorescence inductions data, as stated in Equation (3). This is a double normalization of the fluorescence induction curve that permits a comparison of fluorescence transient measured under different conditions and/or within different samples:

(1) $$F_{V}=F_{M}-F_{O}$$

(2) $$\varphi_{Po}=\frac{F_{V}}{F_{M}}=1-\frac{F_{O}}{F_{M}}$$

(3) $$V(t)=\frac{\left(F(t)-F_{O}\right)}{\left(F_{M}-F_{O}\right)}$$

The above processes are applied to photosynthetic samples that have been kept in darkness, but, for samples that are kept in light, there are variants of this analysis. These measurements are commonly made during the decrease stage of fluorescence induction, utilizing a pulse of intense light between P and S time intervals as is highlighted in Figure 1. This leads to a transient increase of the fluorescence to a maximum value that, for the samples kept in light, this is called F'_M_. This is usually lower than the F_M_ measured in dark-adapted samples. This decrease of maximum fluorescence during the slow phase of fluorescence induction is attributed to the non-photochemical process [42]. Similarly, the F_O_ value of the sample kept in light is labeled as F'_O_, being slightly different [43]. Nevertheless, the change is small enough to be negligible and it can be assumed that F_O_ = F'_O_.

To calculate the non-photochemical quenching of samples kept in light, the expression in Equation (4), is used [44]. As has been observed, it is not necessary to differentiate the F_O_ and F'_O_ values. However, it is noted that F'_M_ is affected by photochemical quenching too. There are definitive results with samples that had been kept in darkness, and these can serve as reference for the normalization of data obtained from samples that have been kept in light. In such cases the formula for non-photochemical quenching is Equation (5). Taking into consideration that F'_O_ = F_O_, the non-photochemical quenching formula for light-kept samples becomes Equation (6), whereas the photochemical quenching formula for light-kept samples is expressed as Equation (7). It must be taken into account that q_N_ and q_P_ are not two complementary terms, meaning that q_N_ and q_P_ are not equal to one [33]:

(4) $$\frac{\left(F_{M}-{F\prime }_{M}\right)}{{F\prime }_{M}}$$

(5) $$q_{N}=1-\frac{\left({F\prime }_{M}-{F\prime }_{O}\right)}{\left(F_{M}-F_{O}\right)}$$

(6) $$q_{N}=\frac{\left(F_{M}-{F\prime }_{M}\right)}{\left(F_{M}-F_{O}\right)}$$

(7) $$q_{P}=1-\frac{\left({F\prime }_{M}-F(t)\right)}{\left({F\prime }_{M}-{F\prime }_{O}\right)}$$

To begin with, the first instrumentation systems for chlorophyll fluorescence measurement evolved slowly. This happened because there was not much variety in the electronic devices available to develop these instruments, limiting possible research projects for lack of suitable technology. For example, in earlier tests, quartz fluorescence tubes with lens, prisms and photoelectric cells had to be used to acquire the fluorescence response. Today, far more advanced instrumentation and new technologies are available in order to provide a light source for this procedure.

## 3. Instrumentation in Chlorophyll Fluorescence

Most applications of chlorophyll fluorescence are focused on herbaceous plants, as these represent the main part of agricultural production. There are many kinds of measurements for monitoring plant variables, but these methods always attempt to be less invasive; that is, to make the measurements without causing damage and with a minimum amount of stress to the plant. This is why the chlorophyll fluorescence method is so successful. The instrumentation methods used to carry this out are thought to be the least invasive possible, even when the measurements are made in the laboratory.

**Figure 2.**
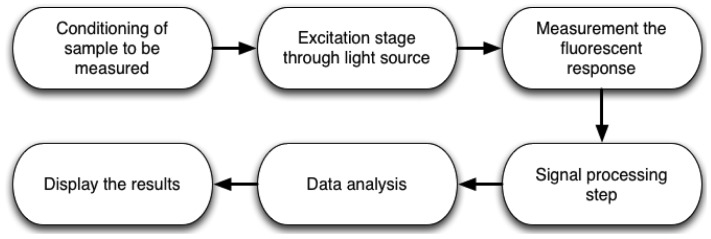
Stages of chlorophyll fluorescence measurement process.

The complete methodology to measure chlorophyll fluorescence is shown in Figure 2. First, it is necessary to prepare the sample to be measured. For example, if the measurement is carried out in dark-adapted conditions, the sample must be put inside a light isolation chamber to avoid ambient light interference. Sometimes, microorganisms need pre-treatments to improve the measurement and avoid stress conditions. The next step is the development of system instrumentation, using lamps, LEDs, lasers, among others as excitation light source on active methods. However, there are passive methods which utilize only sunlight. This can be an advantage when the designer wants to save on light source devices, but it commonly requires more processing capabilities. The next stage consists of designing and selecting the proper optical detection devices, to obtain the fluorescence response from the previously excited sample for subsequent data storage and analysis. Later, it is necessary to process the response by applying signal processing techniques and displaying data in order to analyze the conditions of the sample.

**Figure 3.**
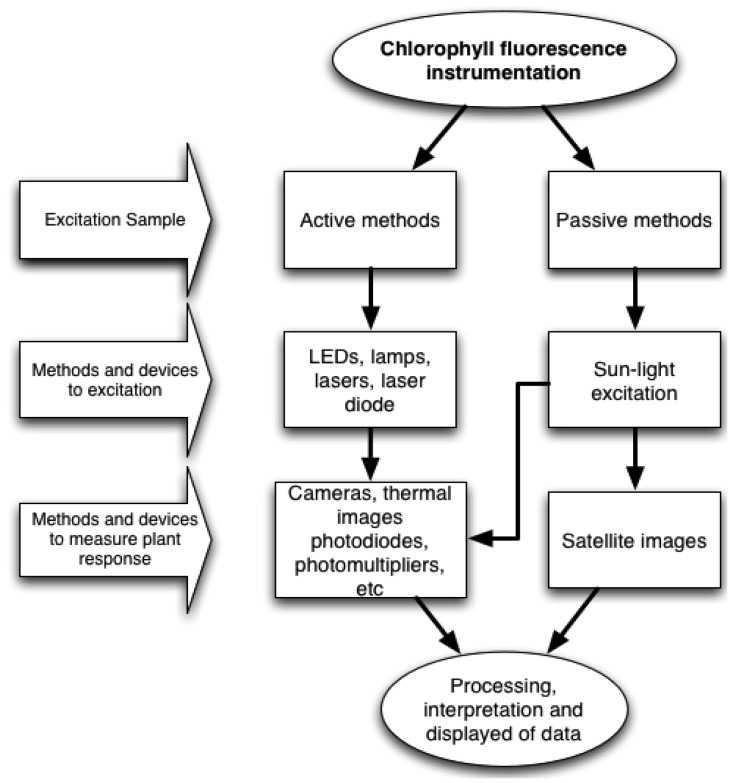
Chlorophyll fluorescence measurement routes by excitation source.

Figure 3 shows two possible ways to perform the measurement process. Active methods are those in which excitation light is provided by electronic devices such as LEDs, lamps, lasers, laser diodes, *etc.* On the other hand, passive methods, utilize only the sunlight energy as an excitation source. Furthermore, there are different ways to obtain the fluorescence response, depending on the excitation method and sample conditions. This can be achieved by using photodetector devices such as, cameras, photodiodes, photomultipliers, among others. It is necessary to process the collected data in order to obtain the chlorophyll fluorescence response to perform a proper interpretation and display results.

### 3.1. Light Sources for Excitation of Chlorophyll

As mentioned before, chloroplasts are very sensitive to stress. Consequently, the instrumentation applied to the plant for chlorophyll excitation should affect as little of the plant as possible. The first objective of study is the excitation light source. Correct selection is critical. First, taking into consideration the stress it is inflicting on the study subject, and second, the kinds of energy being emitted by the light source. It is well known that light sources are not very efficient devices, and often waste too much energy on others types of radiations, such as heat. The chlorophyll needs high light intensity to be activated and submit a response, and herein lays the problem. If the light source is intense, this could cause a harmful increase in temperature, especially when the subject is isolated via a dark chamber [6,18,22].

An appropriate light source must be selected. The first consideration for any light source is a lamp; yet, common lamps emit light primarily through filaments, which are low-efficiency devices due to the wasted energy in the form of heat and infrared emissions. Nevertheless, some kinds of lamps are widely used for plant growth. For example, high-pressure sodium lamps likes Grolux [17] do not use filament, but instead function through the excitation of the gas inside them. However, this type of lamp, like the filament lamp, emits heat that can cause stress to the plant; consequently, causing a disruption in the sample measurement.

New and more efficient alternatives for lighting are constantly being generated; one of the most current is the *Light Emitter Diode* (LED). The LED commonly takes up less space, generates less heat, and has a higher efficiency. This technology is being used regularly as a chlorophyll fluorescence source [6,9,20–25].

Some applications do not need to excite the entire subject, only a small part of it. In these cases lasers or laser diodes are an excellent choice, taking advantage of their special characteristic of emitting coherent light. This means that all of the electromagnetic waves emitted by the laser are pointed in a single direction. In contrast to what occurs with a common lamp where the emissions are dispatched in all directions, also referred to as omnidirectional. The coherent light emitted by these devices is quite a handy feature because a considerable amount of energy can be directed to a small area. This can cut down on the amount of necessary devices, and in some cases even lower costs. Also, these are a good choice for implementing portable chlorophyll fluorescence sensing systems. Such positive attributes are what makes the laser a frequently used device in these applications [16].

**Figure 4.**
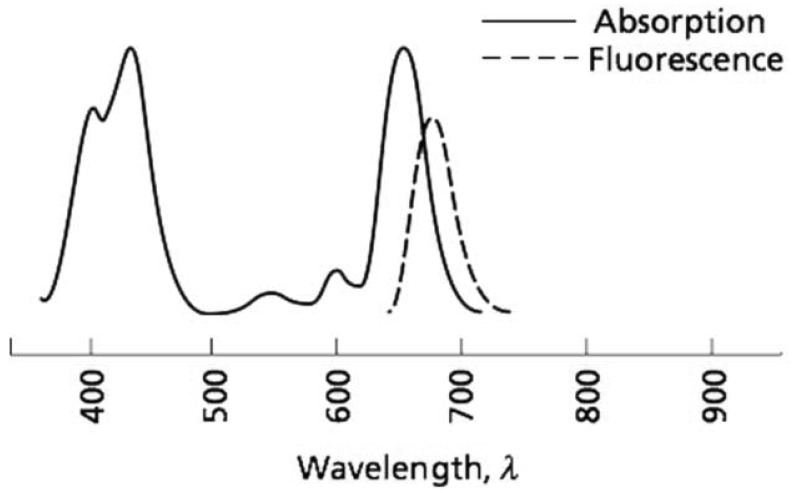
Light absorption and emission by chlorophyll (modified from [1]).

Another important factor to consider for the application of chlorophyll excitation is the wavelength. It is critical to choose the correct excitation wavelength to get the expected response. It is common knowledge that chlorophyll absorbs blue and red wavelengths, but reflects green, which is why the human eyes perceive plants in this hue [1]. The chlorophyll absorption zone on the electromagnetic spectrum and its fluorescence wavelength response are shown in Figure 4. Despite this standard, each chlorophyll excitation application uses a specific wavelength. This is because depending on what intensity or spectrum is the excitation source, the subject will have a different response.

Regarding the correct choice of wavelength, different selections are considered depending on the applications or electronic devices that were used in carrying out the experiment. This is based on the fact that the fluorescence of the plant is a re-emitting process of the absorbed photons with increased wavelength [1]. For example, for a plant that absorbs light in the blue (440 nm) and red (660 nm) wavelengths the chlorophyll fluorescence will manifest as a red and infrared wavelength approximately 700 nm. This happens because energy is lost through heat, as seen in Figure 4.

Depending on the target applications, researchers select different wavelengths for excitation, as well as the appropriate light source, to carry out the fluorescence measurement. Table 1, shows a revision of the wavelengths utilized to excite samples, via various chlorophyll fluorescence sensing systems. Most are in the range from the ultra-violet to infrared.

### 3.2. Detection Devices for Chlorophyll Fluorescence

After the proper selection of chlorophyll excitation light-emitting devices and wavelengths, the second step is to have a way of identifying the response of the excited plant. This is achieved through different methods or sensing devices, depending on the system's design. For example, sometimes when an isolation chamber is used, optical fibers may be needed to convey the plant response to be processed outside of the chamber [14,22,24]. Photodiodes are widely used in these applications, often because they have both a good response time and are low-cost optical sensing devices along with other options for detecting the fluorescence (e.g., photomultipliers, thermal cameras, interferometry, *etc.*). As was aforementioned, photomultipliers are also used [45,46]. These are optical detectors like photodiodes, but with a base of vacuum tubes, and are particularly sensitive to low radiation.

However, the fluorescent response is not always acquired and processed as a one-dimensional signal. On many occasions image processing is also applied, and this is where the use of cameras comes in [19,26]. Generally, researchers tend to be more familiar with cameras as the sensing devices, because it is easy to understand how cameras can capture the response of the excited plant, not only on the visible spectrum but also through thermal imaging. It is possible that the use of thermal imaging is not taking fluorescence into account, only the temperature of the plant. But one must remember that visible light makes up just a small part of the electromagnetic spectrum, and that thermal emissions are located just below the visible, in the infrared spectrum. Taking this into account, thermal cameras are just as common and necessary as those that process visible light, the only difference being that they exhibit greater sensitivity to longer wavelengths.

The application of cameras in this field sometimes has different objectives than those of two-dimensional imaging. For instance, there are camera-based applications that can be used in the quality-control area, such as the research carried out by Lefcourt *et al.* in 2005 [30]. In this study, special filters and edge detections were found to be able to detect when a fruit, in this case a red-delicious apple, was contaminated by fecal waste. In other cases, camera application can be used to detect pathogens even when there are no symptoms visible to the human eye.

Image processing for chlorophyll fluorescence is not only used for small-subject analysis; it is also widely applied for large-scale analysis, as in cases where it is necessary to analyze the fluorescence of high plants, or even large sets of plants, via satellite imaging.

## 4. Measurement Methods for High Plants

It tends to be more difficult to make measurements for high plants, or in other words trees, because they are less manageable than herbaceous plants for either laboratory or field measurements. Therefore, it is sometimes impossible to use the aforementioned techniques for this type of procedure.

This section of the review will focus on the techniques that differ from those used for herbaceous plants. However, it is important to note that some of the chlorophyll fluorescence sensing systems are used in a similar fashion, especially with commercial products such as PEA Hansatech Instruments Ltd., which is used in applications with high as well as herbaceous plants [10].

Some of the methods used in chlorophyll excitation are called passive since they do not use an excitation device, the only excitation source employed being natural sunlight. This can lead to problems, since most of the active measurements are performed under controlled conditions; for example, the sample isolation chamber used in many laboratory measurements. However, when using the sun as a source of chlorophyll excitation the main problem lies in the photon emission response from the plant. This is below that which is emitted by the sun, less 1% [28].

To perform these types of measurements with environmental noise present, different methods have been developed to combat solar interference and obtain an accurate response from the plant. One way to solve this problem is through Fraunhofer lines. This technique is based on the capacity of gases in the atmosphere to absorb certain wavelengths. This means that band-reject filters can be used in such a manner that when determined by the incursion of gas, they can suppress an absorbed wavelength [13,28,47].

This technique can exploit oxygen capability by suppressing the 688 nm and 760 nm wavelengths, which is the bandwidth where chlorophyll fluorescence occurs [11]. Through the suppression of these wavelengths the fluorescence emitted by the plant can be viewed in order to make measurements.

Another way to make measurements on high plants is through the use of satellite [48] or airborne images [49], sometimes even both [50]. In contrast to ground applications, the estimation of chlorophyll fluorescence from air or space-borne sensors complicates the results. Consequently, the chlorophyll fluorescence, which is emitted by plants, needs to be separated from the reflected light by the sample and the atmosphere path radiance conditions, which creates accuracy problems on the sensor [29]. These techniques, due to their nature, are applied to large numbers of subjects at the same time, even on a global level. Because a single subject emitting chlorophyll florescence is negligible using this method, such techniques can be used for large-scale environmental and ecological analysis. They can also be used for the analysis of photosynthesis in primary sector production, because it is well known that these variables are highly related. The methods of chlorophyll excitation for these applications are always passive, due to the nature of their collective analysis.

## 5. Measurement Methods for Micro Organisms

There are other applications of this process contrasting greatly with the mega-analysis methods covered in the previous section; these processes focus on analyzing microorganisms such as phytoplankton, algae, and bacteria. Method-wise, however, these procedures are not significantly different since photosynthetic reactions occur at a cellular level. Some of the commercial tools used to make the fluorescence measurements are actually used in both whole plants and unicellular organisms [50–53].

Despite the similarities in the chlorophyll excitation process and fluorescence response acquisition in both single cells and whole plants, a serious factor to consider is that the manipulation of samples changes noted for microorganisms, as compared to leaf-level measurements. Single-cell samples need special consideration and complex treatment. For example, there is a custom spectrometer modified to carry out measurement of the delayed fluorescence spectra *of Chlorella vulgaris* [52]. In conjunction with the commercial PAM-2000, it is possible to measure the efficiency of photochemical energy conversion in PSII reaction centers in microorganism samples [43].

Another sample type with similar features is algae. One specific case, in an experiment carried out by Drinovec in 2011 [54], the delayed fluorescence excitation spectra were measured with a custom-built delayed fluorescence spectrometer. The excitation was performed by halogen lamp and linear filter, while light was detected with a Perkin Elmer C1393 channel photomultiplier in photon-counting mode, assisted by two electromagnetic shutters used to protect the photomultiplier from the excitation light. This is because, as was aforementioned, the photomultiplier is a highly sensitive device and is subsequently used in lower light conditions.

Cyanobacteria are another type of microorganism capable of being analyzed via these methods. Cyanobacteria are thought, evolutionarily speaking, to be among the oldest organisms; they can perform oxygenic photosynthesis and respiration simultaneously, and many species are able to fix nitrogen. Within cyanobacteria similar conditions can be found and reactions such as those found in plants; for example, stress caused by heavy metal effects. These kinds of stress conditions can be detected by the previously detailed commercial methods, but these need to have been previously modified for this application. The container, especially, is different in this context. A cuvette equipped with sensors, such as piezoelectric transducers attached to its wall, usually serves as a detection system [53].

Wavelengths for chlorophyll excitation and subsequent fluorescence responses are not much different in analysis, between plants and microorganisms. However, it is necessary to perform tests to determine the most appropriate wavelength to use with any appliance, because the fluorescence excitation spectrum of fluorescence depends on the various cell pigments [52]. It is even possible to take advantage of this situation, since samples in different taxonomic groups can be discriminated using delayed fluorescence spectroscopy by applying a sweep of wavelength, for example in a range between 400 nm and 700 nm [53].

## 6. Chlorophyll Fluorescence Signal Processing and Drivers

In this review the methods and electronic devices to elicit the chlorophyll fluorescence as well as the measurement of the response have been analyzed. Nevertheless, none of these are functional without data or signal processing, with the overall goal of estimating the condition or characteristics of the sample being measured.

Some equipment just displays the fluorescence behavior graphs and the operator is responsible for interpreting those graphs. Yet, other equipment is able to process information to show clear results such as photochemical and non-photochemical quenching. Depending on the pretreatment of the sample, the formula is selected like light-adapted or dark-adapted quenching [33].

Commercial equipment, generally known as PAM, functions with a Pulse Amplitude Modulated and operates without isolation chambers, which means that it is susceptible to environmental noise. In these cases, it is necessary to separate the fluorescent plant response in sunlight from fluorescence caused by chlorophyll excitation device. This is achieved by the pulse modulation of light source device, which induces a pulsed chlorophyll fluorescence signal form, the sample within conditions where ambient light is excluded with the assistance of optical filters permitting only three types of light signals: ambient light, non-pulsed fluorescence signal induced by the ambient light, and pulsed fluorescence signal induced by the modulation light source. It is noteworthy that this process is an active method, because the source light is different from the sun.

**Figure 5.**
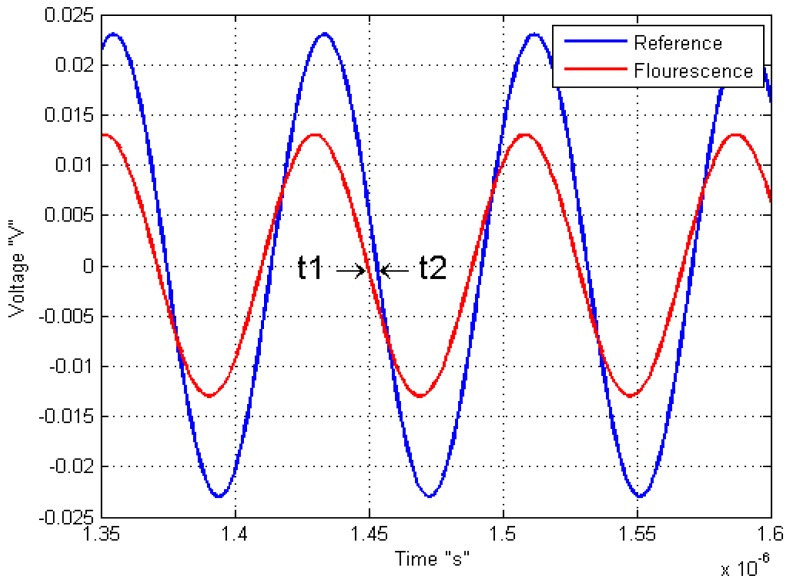
Sine waveform of light source chlorophyll excitation with a fluorescent response (Modified of [21]).

Another example of modified light source is used by Kissinger and Wilson on 2011, where they connected an oscillator to vary the LED source, with the objective to know the response of the sample by a sine-modulated excitation. This means that the phase difference between the reference or signal excitation and the output signal of the sample response indicates the fluorescence lifetime response as shown on Figure 5.

There are a variety of commercial or custom instruments to measure fluorescence, all of these aim to be less expensive and more accurate than other complex and inefficient methods. The fluorescence measurement has many advantages compared with other photosynthesis measurement methods. Chlorophyll fluorescence has the advantages of low energy consumption, fast measurement process, portability, lower cost, to name but a few when it is compared with carbon dioxide exchange method.

## 7. Main Applications of Chlorophyll Fluorescence

Chlorophyll fluorescence is a defense mechanism or excess energy dissipation performed by the subject [33] that is highly linked to the photosynthetic process. Consequently, it is one of the main applications used to estimate the photosynthesis level. Considering photosynthesis maintains autotrophic life, it is imperative to seek new alternatives for its measurement estimations. Chlorophyll florescence is one of them.

The main reason behind of these chlorophyll fluorescence sensing systems is to determine the comfort status of the plant, which gives one an idea of how productive the plant will become. However, these comfort conditions are not only reflected in the photosynthesis estimate, but also in the measurement of chlorophyll fluorescence. This means that it is not necessary to perform photosynthesis estimation procedures, because stress can also be detected through the sole use of chlorophyll fluorescence measurement. The quenching curves alone reflect several types of stress, including hydric or drought [55,56], frost damage [57], ozone [8], nutritional (such as boron) [15] and nitrogen [16,27,47].

There are other interesting features that can be detected by fluorescence as well; one being the amount of anthocyanins and flavonoids present in a subject. These can be detected by the surface skin fluorescence, as has been done with grapes [58]. Such uses are not as common as stress detection but are a good alternative to avoid destructive methods in these types of processes.

A method of identifying traces of feces in food was previously discussed. This takes advantage of the bacteria or plant remains found in feces, which are made to fluoresce. Through filters and image processing, the contaminating material can be detected. This can be applied to evaluate fruit washing methods, and therefore result in cleaner food without risk of disease [30]. As can readily be seen, the chlorophyll fluorescence method is a heavily exploited and is a useful tool in the areas of industry, agriculture, food, and research, including the area of global climate change.

## 8. Conclusions

The main advantage of chlorophyll fluorescence measurement is that it has the possibility of being a non-invasive technique. This differs from other stress-detection methods in which the measurement itself causes undue stress to the plant, which in turn can alter the resulting measurements. Based on these facts, many techniques for chlorophyll fluorescence measurement have been developed with the aim of improving comfort conditions for the plant and of obtaining increased production rates. However, these are not the only applications, because significant research on climate and environmental impact can also be carried out via chlorophyll fluorescence, which can be measured with the use of satellite images or manual methods. Due to various ecological problems, this is now more important than ever before.

Taking into account that electronic devices are constantly evolving and becoming more efficient, equipment to measure chlorophyll fluorescence will constantly improve; therefore, making smaller, more precise and more accurate measurements possible. However, there are many pathways to create new biosensors research. Therefore, this article review should be helpful in achieving a wide overview of state of the art investigations. For example, new methods and waveforms in the excitation light source could be utilized with the goal of finding new expressions in chlorophyll fluorescence response, and enhancing the methods of detecting bad conditions or problems in samples.

In modern fluorescence biosensing systems, it is necessary to perform the measurement in real time, with an optimal resolution and with portable biosensing systems. Furthermore, the aim is to improve the detection of stress conditions on plants of interest, as well as creating automated and easier measurement methods, in order to perform a large number of measurements at the same time without requiring highly qualified personnel. To carry out real-time measurement, it is necessary to design embedded sensor systems and reduce dependency on stationary and manual laboratory equipment.

However, none of these applications can be carried out without the appropriate instrumentation or equipment. As such, this review has been written with the goal of both widening general knowledge of these kinds of applications, and of providing the necessary information to develop novel and custom biosensors for chlorophyll fluorescence measurement.

## Figures and Tables

**Table 1.** Wavelength use rate in chlorophyll sample excitation.

**Wavelength** **(nm)**	**365**	**375**	**400**	**450**	**460**	**470**	**477**	**520**	**528**	**454**	**580**	**600**	**628**	**630**	**640**	**650**	**655**	**660**	**665**	**700**	**740**
Use rate	1	1	1	1	1	1	1	1	1	1	1	1	1	1	1	3	1	2	1	1	1

## References

[1] Taiz L., Zeiger E. (2002). Plant Physiology.

[2] Long S.P., Humphries S., Falkowski P.G. (1994). Photoinhibition of photosynthesis in nature. Annu. Rev. Plant. Mol. Biol..

[3] Losciale P., Hendrickson L., Grappadelli L.C., Chow W.S. (2011). Quenching partitioning through light-modulated chlorophyll fluorescence: A quantitative analysis to assess the fate of the absorbed light in the field. Environ. Exp. Bot..

[4] Zarco-Tejada P.J., Miller J.R., Mohammed G.H., Noland T.L., Sampson P.H. (2002). Vegetation stress detection through chlorophyll a + b estimation and fluorescence effects on hyperspectral imagery. J. Environ. Qual..

[5] Genty B., Briantais J.M., Baker N.R. (1989). The relationship between the quantum yield of photosynthetic electron transport and quenching of chlorophyll fluorescence. Biochim. Biophys. Acta.

[6] Fedack V., Kytaev O., Klochan P., Romanov V., Voytovych I. Portable Chronofluorometer for Express-Diagnostics of Photosynthesis.

[7] Espinosa-Calderon A., Torres-Pacheco I., Padilla-Medina J.A., Osornio-Rios R.A., Romero-Troncoso R.J., Villaseñor-Mora V., Rico-Garcia E., Guevara-Gonzalez R.G. (2011). Description of photosynthesis measurement methods in green plants involving optical techniques, advantages and limitations. Afr. J. Agric. Res..

[8] Bussotti F., Desotgiu R., Cascio C., Pollastrini M., Gravano E., Gerosa G., Marzuoli R., Nali C., Lorenzini G., Salvatori E. (2011). Ozone stress in woody plants assessed with chlorophyll a fluorescence. A critical reassessment of existing data. Environ. Exp. Bot..

[9] Faraloni C., Cutino I., Petruccelli R., Leva A.R., Lazzeri S., Torzillo G. (2011). Chlorophyll fluorescence technique as a rapid tool for *in vitro* screening of olive cultivars (*Olea europaea* L.) tolerant to drought stress. Environ. Exp. Bot..

[10] Mereu S., Gerosa G., Marzuoli R., Fusaro L., Salvatori E., Finco A., Spano D., Manes F. (2011). Gas exchange and JIP-test parameters of two Mediterranean maquis species are affected by sea spray and ozone interaction. Environ. Exp. Bot..

[11] Colls J.J., Hall D.P. (2004). Application of a chlorophyll fluorescence sensor to detect chelate-induced metal stress in Zea mays. Photosynthetica.

[12] Contreras-Medina L.M., Torres-Pacheco I., Guevara-González R.G., Romero-Troncoso R.J., Terol-Villalobos I.R., Osornio-Rios R.A. (2009). Mathematical modeling tendencies in plant pathology. Afr. J. Agric. Res..

[13] Frankenberg C., Fisher J.B., Worden J., Badgley G., Saatchi S.S., Lee J.E., Toon J.C., Butz A., Jung M., Kuze A. (2011). New global observations of the terrestrial carbon cycle from GOSAT: Patterns of plant fluorescence with gross primary productivity. Geophys. Res. Lett..

[14] Piccotto M., Bidussi M., Tretiach M. (2011). Effects of the urban environmental conditions on the chlorophyll a fluorescence emission in transplants of three ecologically distinct lichens. Environ. Exp. Bot..

[15] Guidi L., Degl'Innocenti E., Carmassi G., Massa D., Pardossi A. (2011). Effects of boron on leaf chlorophyll fluorescence of greenhouse tomato grown with saline water. Environ. Exp. Bot..

[16] Thoren D., Schmidhalter U. (2009). Nitrogen status and biomass determination of oilseed rape by laser-induced chlorophyll fluorescence. Eur. J. Agron..

[17] Van Gaalen K.E., Flanagan L.B., Peddle D.R. (2007). Photosynthesis, chlorophyll fluorescence and spectral reflectance in Sphagnum moss at varying water contents. Oecologia.

[18] Hideg E., Schreiber U. (2007). Parallel assessment of ROS formation and photosynthesis in leaves by fluorescence imaging. Photosynth. Res..

[19] Lichtenthaler H.K., Langsdorf G., Lenk S., Buschmann S. (2005). Chlorophyll fluorescence imaging of photosynthetic activity with the flash-lamp fluorescence imaging system. Photosynthetica.

[20] Johnson X., Vandystadt G., Bujaldon S., Wollman F.A., Dubois R., Roussel P., Alric J., Béal D. (2009). A new setup for *in vivo* fluorescence imaging of photosynthetic activity. Photosynth. Res..

[21] Kissinger J., Wilson D. (2011). Portable fluorescence lifetime detection for chlorophyll analysis in marine environments. IEEE Sens. J..

[22] Wang J., Xing D., Zhang L., Jia L. (2007). A new principle photosynthesis capacity biosensor based on quantitative measurement of delayed fluorescence *in vivo*. Biosens. Bioelectron..

[23] Avercheva O.V., Berkovich Y.A., Erokhin A.N., Zhigalova T.V., Pogosyan S.I., Smolyanina S.O. (2009). Growth and photosynthesis of chinese cabbage plants grown under light-emitting diode-based light source. Russ. J. Plant. Phys..

[24] Bulgarea G., Boukadoum M. (2001). A high-performance instrumentation system to measure the fluorescence kinetics of plants for *in vivo* photosynthesis research. IEEE. T. Instrum. Meas..

[25] Ji J.W., Xu M.H., Li Z.M. (2010). Research and application on chlorophyll fluorescence on-line monitoring technology. Adv. Mat. Res..

[26] Kolber Z., Klimov D., Ananyev G., Rascher U., Berry J., Osmond B. (2005). Measuring photosynthetic parameters at a distance: Laser induced fluorescence transient (LIFT) method for remote measurements of photosynthesis in terrestrial vegetation. Photosynth. Res..

[27] Schächtl J., Huber G., Maidl F.X., Sticksel E., Schulz J., Haschberger P. (2005). Laser-induced chlorophyll fluorescence measurements for detecting the nitrogenstatus of wheat (*Triticum aestivum* L.) canopies. Prescis. Agric..

[28] Liu L., Zhang Y., Wang J., Zhao C. (2005). Detecting solar-induced chlorophyll fluorescence from field radiance spectra based on the fraunhofer line principle. IEEE Trans. Geosci. Remote.

[29] Meroni M., Rossini M., Guanter L., Alonso L., Rascher U., Colombo R., Moreno J. (2009). Remote sensing of solar-induced chlorophyll fluorescence: Review of methods and applications. Remote. Sens. Environ..

[30] Lefcourt A.M., Kim M.S., Chen Y. (2005). A transportable fluorescence imagining system for detecting fecal contaminants. Comput. Electron. Agric..

[31] Agati G., Cerovic Z.G., Pinelli P., Tattini M. (2011). Light-induced accumulation of ortho-dihydroxylated flavonoids as non-destructively monitored by chlorophyll fluorescence excitation techniques. Environ. Exp. Bot..

[32] Kautsky H., Hirsch A. (1931). Neue versuche zur kohlensäureassimilation. Naturwissenschaften.

[33] Stirbet A., Govindjee (2011). On the relation between the Kautsky effect (chlorophyll a fluorescence induction) and photosystem II: Basics and applications of the OJIP fluorescence transient. J. Photochem. Photobiol. B.

[34] Lin Z., Liu N., Lin G., Pan X., Peng C. (2007). Stress-induced alteration of chlorophyll fluorescence polarization and spectrum in leaves of *Alocasia macrorrhiza* L. Schott. J. Fluoresc..

[35] Sun Z., Lee H., Matsubara S., Hope A.B., Pogson B.J., Hong Y., Chow W.S. (2006). Photoprotection of residual functional photosystem II units that survive illumination in the absence of repair, and their critical role in subsequent recovery. Physiol. Plant.

[36] Hendrickson L., Förster B., Pogson B.J., Chow W.S. (2005). A simple chlorophyll fluorescence parameter that correlates with the rate coefficient of photoinactivation of photosystem II. Potosynth. Res..

[37] Campbell D., Hurry V., Clarke A.K., Gustafsson P., Öquist G. (1998). Chlorophyll fluorescence analysis of cyanobacterial photosynthesis and acclimation. Microbiol. Mol. Biol. Rev..

[38] Buonasera K., Lambreva M., Rea G., Touloupakis E., Giardi M.T. (2011). Technological applications of chlorophyll a fluorescence for the assessment of environmental pollutants. Anal. Bioanal. Chem..

[39] Björkman O., Demmig B. (1987). Photon yield of O_2_ evolution and chlorophyll fluorescence characteristics at 77 K among vascular plants of diverse origins. Planta.

[40] Govindjee (1995). Sixty-three years since Kautsky: Chlorophyll a fluorescence. Aust. J. Plant Physiol..

[41] Govindjee (2004). Chlorophyll A Fluorescence: A Bit of Basics and History. Chlorophyll a Fluorescence A Signature of Photosynthesis.

[42] Krause G.H., Jahns P., Papageorgiou G.C., Govindjee (2004). Non-Photochemical Energy Dissipation Determined by Chlorophyll Fluorescence Quenching: Characterization and Function. Chlorophyll a Fluorescence: A Signature of Photosynthesis (Advances in Photosynthesis and Respiration).

[43] Bilger W., Schreiber U. (1986). Energy-dependent quenching of dark level chlorophyll fluorescence in intact leaves. Photosynth. Res..

[44] Bilger W., Björkman O. (1990). Role of the xanthophyll cycle in photoprotection elucidated by measurements of light-induced absorbance changes, fluorescence and photosynthesis in leaves of Hedera canariensis. Photosynth. Res..

[45] Bürling K., Hunsche M., Noga G. (2011). Use of blue-green and chlorophyll fluorescence measurements for differentiation between nitrogen deficiency and pathogen infection in winter wheat. J. Plant Physiol..

[46] Hunsche M., Bürling K., Noga G. (2011). Spectral and time-resolved fluorescence signature of four weed species as affected by selected herbicides. Pestic. Biochem. Phys..

[47] Zhang Y., Liu L., Hou M., Liu L., Li C. (2009). Progress in remote sensing of vegetation chlorophyll fluorescence. J. Remote Sens..

[48] Xing X., Zhao D., Liu Y., Yang J., Xiu P., Wang L. (2007). An overview of remote sensing of chlorophyll fluorescence. Ocean Sci. J..

[49] Zarco-Tejada P.J., González-Dugo V., Berni J.A.J. (2012). Fluorescence, temperature and narrow-band indices acquired from a UAV platform for water stress detection using a micro-hyperspectral imager and a thermal camera. Remote Sens. Environ..

[50] Koponen S., Attila J., Pulliainen J., Kalliob K., Pyhälahtib T., Lindfors A., Rasmus K., Hallikainen M. (2007). A case study of airborne and satellite remote sensing of a spring bloom event in the Gulf of Finland. Cont. Shelf Res..

[51] Kurzbaum E., Eckert W., Yacobi Y.Z. (2007). Delayed fluorescence as a direct indicator of diurnal variation in quantum and radiant energy utilization efficiencies of phytoplankton. Photosynthetica.

[52] Kurzbaum E., Beer S., Eckert W. (2010). Alterations in delayed and direct phytoplankton fluorescence in response to the diurnal light cycle. Hydrobiologia.

[53] Dudkowiak A., Olejarz B., Łukasiewicz J., Banaszek J., Sikora J., Wiktorowicz K. (2011). Heavy metals effect on cyanobacteria synechocystis aquatilis study using absorption, fluorescence, flow cytometry, and photothermal measurements. Int. J. Thermophys..

[54] Berden-Zrimec M., Drinovec L., Zrimec A., Suggett D.J., Prášil O., Borowitzka M.A. (2010). Delayed Fluorescence. Developments in Applied, Chlorophyll Fluorescence in Aquatic Science: Methods and Applications.

[55] Komura M., Yamagishi A., Shibata Y., Iwasaki I., Itoh S. (2010). Mechanism of strong quenching of photosystem II chlorophyll fluorescence under drought stress in a lichen, *Physciella melanchla*, studied by subpicosecond fluorescence spectroscopy. Biochim. Biophys. Acta.

[56] Rahbarian R., Khavari-Nejad R., Ganjeali A., Bagheri A., Najafi F. (2011). Drought stress effects on photosynthesis, chlorophyll fluorescence and water relations in tolerant and susceptible chickpea (*Cicer Arietinum* L.) genotypes. Acta Biol. Crac. Bot..

[57] Rapacz M., Sasal M., Gut M. (2011). Chlorophyll fluorescence-based studies of frost damage and the tolerance for cold-induced photoinhibition in freezing tolerance analysis of triticale (×*Triticosecale* Wittmack). J. Agron. Crop Sci..

[58] Cerovic Z.G., Moise N., Agati G., Latouche G., Ghozlen N.B., Meyer S. (2008). New portable optical sensors for the assessment of winegrape phenolic maturity based on berry fluorescence. J. Food. Compos. Anal..

